# The good, the bad, and the ugly: How to protect chromosome stability from potential threats: A report on the Chromosome Stability Meeting, Bangalore, India, 14‐17 December, 2014

**DOI:** 10.1002/bies.201500023

**Published:** 2015-04-17

**Authors:** Nishant K.T, Kaustuv Sanyal

**Affiliations:** ^1^ School of BiologyIndian Institute of Science Education and ResearchThiruvananthapuramTrivandrumIndia; ^2^ Molecular Biology and Genetics UnitJawaharlal Nehru Centre for Advanced Scientific ResearchBangaloreIndia

## Abstract

Group photo of the participants at the chromosome stability meeting in Jawaharlal Nehru Centre for Advanced Scientific Research (JNCASR), Bangalore, India. The meeting brought together the Indian scientific community and investigators from other countries working on various aspects of chromosome stability.

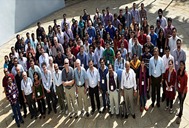

## Introduction

**Figure 1:**
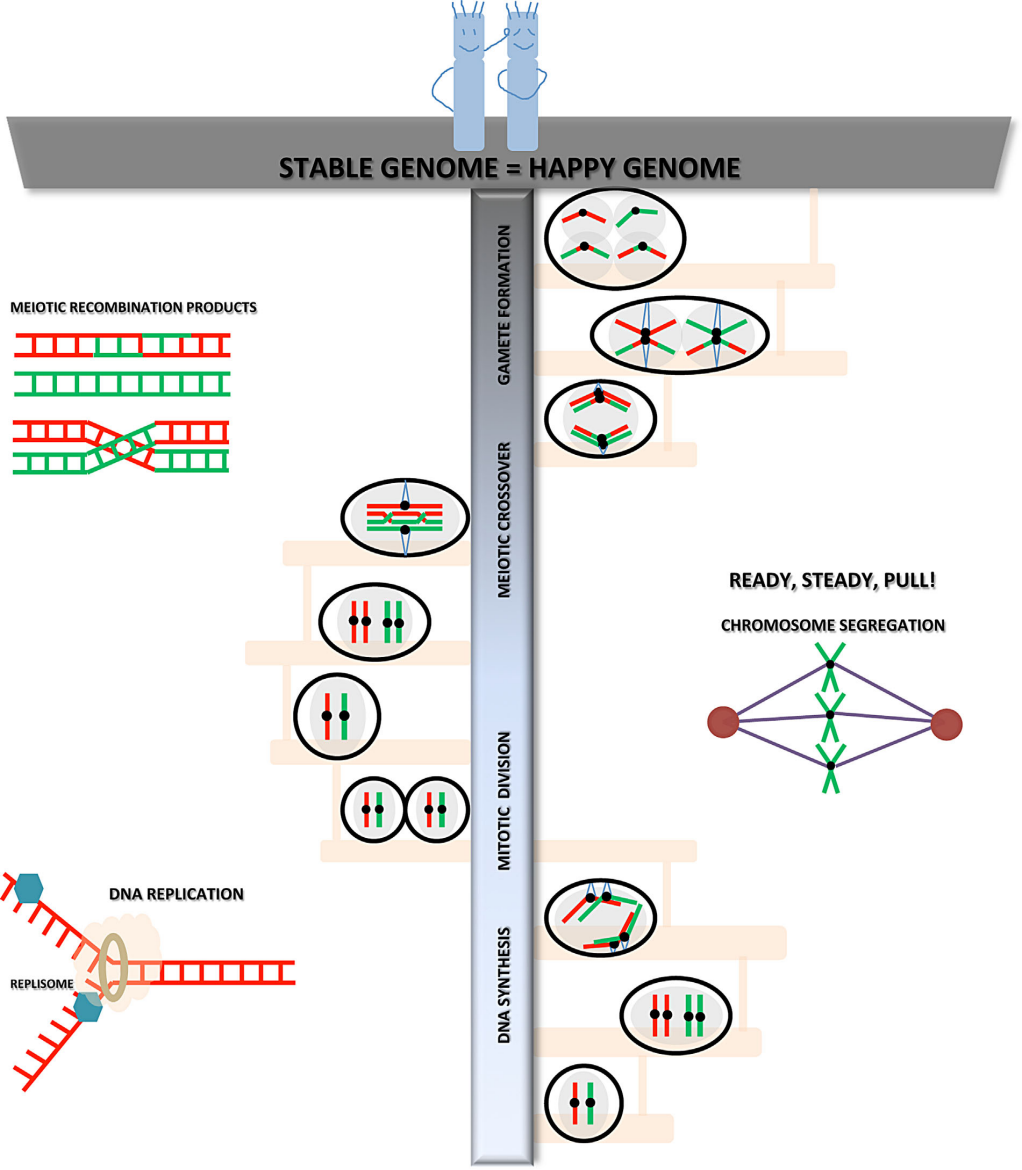
Aspects of chromosome stability during mitosis and meiosis discussed during the meeting. A diploid cell containing a homologous pair of chromosomes (red and green) is shown at the lower most step of the DNA ladder. As a cell progresses through the cell cycle, it undergoes DNA replication to double its chromosomal content. The duplicated set of chromosomes attach to the mitotic spindle and are segregated into two daughter cells. Several talks at the meeting discussed DNA repair and recombination pathways during mitosis as well as centromere evolution and its interaction with the spindle/kinetochore apparatus that ensures the two daughter cells are identical with no genetic variation. In contrast to the mitotic division in somatic cells, germ cells undergo meiotic cell division that starts with a single round of DNA replication followed by two successive rounds of chromosome segregation. During meiosis, recombination between homologous chromosomes facilitates accurate homolog segregation and genetic variation in the gametes. Meiotic chromosome structure and chromatin remodelling, homolog pairing, meiotic recombination pathway choice, and mechanisms of crossover formation were discussed during the meeting. Events occurring in mitosis and meiosis, such as DNA replication, chromosome segregation and meiotic recombination are shown.

The maintenance of chromosome stability depends on the precise duplication of the genome, efficient DNA repair and recombination, and proper interactions between the centromeres/kinetochores of the duplicated chromosomes and the spindle apparatus (Fig. 1). Errors in these processes lead to genomic instability and aneuploidy—a hallmark of many diseases, including cancers. Knowledge of these processes at the molecular level is also essential for understanding principles of genome evolution, organization, and variability. We organized a conference on chromosome stability at the Jawaharlal Nehru Centre for Advanced Scientific Research (JNCASR) in Bangalore, India to facilitate interactions between the fast growing Indian community interested in areas related to chromosome stability with investigators from other countries. The meeting featured talks from 40 principal investigators and postdoctoral fellows representing India, the United States of America (USA), Europe, and Japan. Approximately 80 graduate and undergraduate students from India participated in the meeting. We present here highlights of the meeting.

## DNA repair and recombination pathways

Accurate DNA repair and recombination is critical for chromosome stability. Jim Haber (Brandeis University, USA) discussed genome stability during repair of a broken chromosome in budding yeast. Double‐strand breaks (DSBs) can be repaired by nonhomologous end‐joining (NHEJ) or by homologous recombination in which a intact, identical, or nearly identical sequence is used as a template. However, even conservative repair of DSBs by homologous recombination is associated with a highly elevated risk of mutation. Such mutations often have a “signature” associated with slippage or template switching of the repair DNA polymerases. His group showed that pairs of such inter‐chromosomal template switching events can occur between ectopic (non‐allelic) homologous sequences as often as once every 100 repair events. These types of instability associated with ectopic recombination may underlie some of the complex rearrangements seen in human developmental diseases or in cancer cell chromosomes exhibiting chromothripsis in which individual chromosomes are shattered and then reassembled. Eric Alani (Cornell University, USA) continued with the theme of homologous recombination between diverged DNA sequence in yeast. He discussed how cells make the decision to either unwind and reject recombination intermediates containing mismatches (heteroduplex rejection), or to maintain the intermediates and correct the mismatches. He showed that overexpression of the mismatch repair protein Msh6 can decrease or even stimulate heteroduplex rejection, depending on whether the recombination occurs in the context of a replication fork or independent of it. Thus, even though higher levels of Msh6 may improve recombination fidelity in general, this protein appears to reduce recombination fidelity during replication.

The identification of genomic regions prone to instability due to mitotic homologous recombination is important. Tom Petes (Duke University, USA) described evidence that spontaneous mitotic crossovers in yeast are often initiated by a break in unreplicated DNA and presented genome‐wide mapping of mitotic crossovers in yeast. Analysis of mitotic crossover hotspots showed that they are often associated with closely‐linked inverted repeats. Jennifer Surtees (University at Buffalo, USA) summarized her analysis of the mismatch repair complex (Msh2/Msh3) on expansions of trinucleotide repeat (TNR) tracts in yeast. She showed that msh3 mutations result in a reduction in tract expansions, and proposed a model by which the Msh2/Msh3 complex stabilized small DNA loops to promote tract expansion. These results demonstrate unforeseen consequences of active MMR in the context of TNR tracts and have important implications for understanding diseases caused by TNR expansions.

The above talks discussed the potential of homologous recombination to generate genome rearrangements and instability. Wolf Heyer (University of California‐Davis, USA) discussed how recombination has evolved as a pathway with metastable, reversible intermediates to avoid inappropriate DNA strand invasions in non‐allelic targets and thus minimize the potential for genomic instability. Using in vitro assays, he demonstrated how the anti‐recombination helicase Srs2 dissociates the strand invasion catalysed by Rad51‐ssDNA filaments, whereas Topoisomerase 3 reverses D‐loops exerting anti‐recombination activity. These results suggest that recombination and repair pathways should not be seen as a fixed sequence of events, rather as displaying reversibility at multiple steps. Pathway reversibility creates novel circumstances where synthetic lethality can potentially be exploited in targeting recombination pathways during anti‐cancer therapy. Sathees Raghavan (IISc, India) discussed DNA damage and chromosomal instability in mammalian cells with a focus on the less‐studied mitochondrial DSB repair pathway. He showed that Microhomology Mediated End Joining (MMEJ) or alternative‐NHEJ are the preferred pathways for rejoining DNA with DSBs in mitochondria.

## DNA repair and recombination in maintenance of genome stability

Defects in DNA repair and recombination pathways cause diseases through genome instability. In the EMBO lecture, Ashok Venkitaraman (Cambridge University, UK) described evidence that tumors arising in carriers that are heterozygous for a tumor suppressor gene are not necessarily the consequence of the “two‐hit” model of Knudson. More specifically, he found that in a murine model for KRAS‐driven pancreatic cancer heterozygous for a mutation in BRCA2, the wild‐type allele remained active in all of the tumors. Similarly, in human patients carrying germline BRCA2 mutations, the wild‐type allele remained active in ∼75% of cases. These results argue that some tumors are a consequence of haplo‐insufficiency or trans‐dominant effects of the mutant allele. Ganesh Nagaraju (IISc, India) in his talk and Kumar Somyajit in his poster described that distinct complexes of mammalian RAD51 paralogs protect nascent DNA at stalled replication forks and mediate replication restart. B. J. Rao (TIFR‐Bombay, India) outlined the non‐random spatial positions of chromosomes in mammalian nuclei and demonstrated dynamic positional changes exhibited by select gene‐rich chromosomes during the DNA damage response. Umesh Varshney (IISc, India) discussed genome stability in Mycobacteria that possess G + C rich genomes. Because of their habitat in the hypoxic environment of host macrophages, their genomes are at increased risk of cytosine deamination (to uracil) and other forms of DNA damage. He showed that contrary to what was expected, there was a remarkable down‐regulation of Ung (a highly conserved class of uracil DNA glycosylases) in *M. tuberculosis* and *M. smegmatis* in response to hypoxia. Misexpression of Ung in *M. smegmatis* using a hypoxia‐responsive promoter decreased C to T mutations but compromised bacterial survival.

## DNA repair and recombination during meiosis

During meiosis, DSBs are deliberately inflicted on the genome to promote shuffling of genetic material by homologous recombination and proper chromosome segregation. Bernard de Massy (Institute of Human Genetics—Montpelier, France) reported ChIP‐seq experiments showing that the zinc finger protein PRDM9 binding sites are not only sites of meiotic H3K4 methylation (associated with DSBs) but also meiotic recombination hotspots, thus confirming that PRDM9 is the primary determinant of where DSB initiation occurs in the mammalian genome. Once the initiating DSBs are formed, chromosomes must encounter the homolog to recombine. This is facilitated by rapid chromosome movements, which require connections between telomeres and the cytoskeleton. Akira Shinohara (Osaka University, Japan) showed in yeast that phosphorylation of Mps3 re‐localizes it from the centrosome to the nuclear envelope during meiosis, facilitating telomere‐led chromosome movement. Meiotic chromosomes are organized into a series of loops that are tethered to a linear axis containing meiosis‐specific chromosomal proteins. As homologs pair, these axes are conjoined into a structure called the synaptonemal complex (SC). The steps of meiotic recombination occur in the context of the axis. K. Muniyappa (IISc, India) described biochemical and structural studies of the conserved chromosome axis protein Hop1, which binds DNA and promotes interactions between pairs of DNA molecules, consistent with a role in coordinating DNA loops in tethered loop‐axis structures. Michael Lichten (NIH, USA) described how the conserved Sgs1(BLM) helicase/Top3‐Rmi1 strand decatenase complex regulates meiotic recombination pathway choice. He showed that the Sgs1/Top3‐Rmi1 complex prevents aberrant recombination intermediate formation and directs meiotic recombination intermediates towards two alternative pathways. These include, early resolution as non‐crossovers, or capture by SC‐associated proteins that promote the regulated stabilization of intermediates and their later resolution into crossovers. Nishant K. T. (IISER Thiruvananthapuram, India) described the impact of mutants of Msh4, one of these SC‐associated proteins, on obligate crossover formation and chromosome segregation. He showed that the obligate crossover is not protected from variations in crossover frequency and analyzed the contribution of achiasmate chromosome segregation mechanisms.

Three presentations focused on the roles for chromatin modification in the regulation of gene expression and progression during meiosis. Paula Cohen (Cornell University, USA) described work on small non‐coding RNAs and their associated Argonaute proteins in regulating gene silencing of the unpaired arms of the X and Y chromosomes during mouse spermatogenesis. Aushaq Bashir Malla (CDFD, India) in his poster showed that IP6K1, a member of the inositol phosphokinase family, is essential for maintaining genome integrity and orchestrating chromatin remodelling during spermatogenesis, and as a consequence this protein is essential for male fertility. Imran Siddiqi (CCMB, India) described meiotic defects in *Arabidopsis* conferred by loss of two important chromatin‐modifying activities: ARP6, a member of the protein complex that loads histone H2aZ onto chromatin; and DUET, a PHD domain‐containing protein that specifically binds dimethlyated H3K4.

Meiotic recombination is not without its hazards, and Lucas Argueso (Colorado State University, USA) described the development of a model system, in budding yeast, to study non‐allelic homologous recombination. The assay uses engineered yeast chromosomes to simulate what transpires in humans when non‐allelic meiotic recombination occurs between large Low Copy Repeats (LCRs), resulting in germline recurrent copy number variation associated with human genomic disorders such as autism.

## Centromeres, kinetochores, and their roles in chromosome segregation

In addition to accurate DNA repair and recombination, chromosome stability also requires proper assembly of physical structures comprising kinetochores on the centromeric DNA. Kaustuv Sanyal (JNCASR, India) in his talk and Neha Varshney in her poster described the utility of working with yeasts other than *S. cerevisiae* (e.g. human pathogenic yeasts) to analyze centromere structure and function. *Candida albicans*, unlike *S. cerevisiae*, harbors unique centromeric regions on every chromosome. When a native centromere is deleted, neo‐centromere formation takes place at a centromere‐proximal region. However, gene conversion occasionally copies an unaltered native centromere to replace the neo‐centromere. CENP‐A loading at these centromeres is linked to replication fork stalling indicating that repair proteins may have a role to play in maintaining centromeres. Joseph Heitman (Duke University, USA) spoke about the role of centromere linkage in the evolution of the *MAT* locus in *Cryptococcus* species from a tetrapolar type to a bipolar type. In *C. amylolentus*, with a tetrapolar *MAT* configuration, both *MAT* loci were found to be centromere‐linked. The presence of similar retro‐transposable elements in all centromeres in most *Cryptococcus* species suggests a role for centromere‐mediated recombination events in this process. Harmit Malik (FHCRC, USA) described how centromeric proteins are rapidly evolving and that most are under positive selection. An illustration of their rapid evolution is that CENP‐A proteins from even closely related species of *Drosophila* fail to function when heterologously expressed in another species.

The talks on centromere function and evolution were followed by talks on the regulation of kinetochore function. Tapas Manna (IISER Thiruvananthapuram, India) in his talk and Geethu Emily Thomas in her poster provided evidence that the EB1 protein decorates the microtubules on the plus end. It does so by interacting with the Ska complex proteins and forming a ring around microtubules in vitro. Ajit Joglekar (University of Michigan, USA) addressed how unattached kinetochores trigger the spindle assembly checkpoint. He showed compelling evidence that the position of spindle assembly checkpoint (SAC) proteins at the kinetochore is important for its activation. The Dam1 kinetochore complex acts as a barrier and does not allow SAC proteins to interact with the spindle pole body component (Spc105) required for SAC activation.

## Perspectives

A better understanding of the molecular mechanisms that determine stable transmission of genetic material over many generations is critical for understanding how diseases such as cancer arise. Several talks in this meeting highlighted how mechanistic insights into the DNA repair and recombination process or dynamic kinetochore‐microtubule interactions during chromosome segregation can improve human health. The meeting provided an important platform for Indian Principal Investigators and students to engage with the international scientific community on themes related to chromosome stability. We expect future sequels of this meeting will further contribute to the growth of this community in India and its integration with the rest of the world.

